# The Effect of Tranexamic Acid on the Outcome of Total Ankle Replacement

**DOI:** 10.7759/cureus.26706

**Published:** 2022-07-09

**Authors:** Mohammed Ali, Abdalla Hassan, Smit Shah, Anjum Rashid, Ashraf Naguib

**Affiliations:** 1 Trauma and Orthopaedics, North Cumbria Integrated Care NHS Foundation Trust, Carlisle, GBR; 2 Trauma and Orthopaedics, The James Cook University Hospital, Middlesbrough, GBR

**Keywords:** wound complication, transfusion rate, postoperative wounds, tranexamic acid, total ankle arthroplasty

## Abstract

Background: Infection rates after total ankle replacement (TAR) are known to be greater than those after hip or knee arthroplasty. Swelling after TAR can make wound healing more difficult, which can lead to infection. Tranexamic acid (TXA) has been shown to minimize blood loss after surgery, improving healing outcomes. We aim to assess the effect of TXA on blood loss and wound complications in TAR.

Methods: The research looked retrospectively at patients who had TAR procedures between September 2014 and December 2019. The procedures were done using the anterior approach at a single hospital by two, foot and ankle surgeons. Tranexamic acid was given intraoperatively before the tourniquet was inflated. The surgeons did not use surgical drains. Pre and postoperative hemoglobin levels, outcome scores as well as post-operative complications were all documented.

Results: A total of 69 patients were included in the study with 33 of them receiving TXA. With a mean age of 67.2, we had 31 females and 38 males. Tranexamic acid was given in doses ranging from 1 gm to 2 gm. None of the patients required blood transfusions after surgery, and there was no statistically significant difference in pre and postoperative hemoglobin levels between the two groups. In the TXA group, there were fewer wound complications. The TXA group achieved better results compared to the non-TXA group (p=0.0130).

Conclusion: Tranexamic acid is safe and effective in lowering postoperative bleeding and preserving hemostasis after deflating the tourniquet, reducing edema and postoperative wound problems such as breakdown and dehiscence.

## Introduction

For a high number of patients with considerable joint damage, total ankle replacement (TAR) has become a viable surgical option [1]. Because it gives a good range of motion and facilitates early mobilization, TAR has been demonstrated to overcome the previous challenges associated with fusion [2]. Furthermore, TAR has a faster rehabilitation course and considerably higher functional outcomes, particularly in young, high-demand patients [3]. However, compared to hip or knee arthroplasty, the incidence of infection, nerve injury, implant loosening, and periprosthetic fracture cannot be regarded as minimal [4]. Infections after total hip or knee replacement have been observed in up to 3% of patients, whereas profound infection rates after total ankle arthroplasty (TAR) has been reported to be as high as 13% in a recent meta-analysis [4,5]. Impaired wound healing can result in a deep infection, which is a life-threatening condition [6]. Insufficient blood flow in the wound margins due to an improper skin incision or severe soft tissue dissection are two common causes of wound healing difficulties.

Furthermore, a large amount of swelling might cause skin necrosis, leading to infection [7]. The management of perioperative blood loss, specifically postoperative hemarthrosis, is a controllable factor affecting patient recovery, complication rates, and hospital expenses, according to a review of the literature on total knee and total hip arthroplasty (TKA) and total hip arthroplasty (THA) [8]. Drain output has been utilized to determine the amount of blood that has accumulated intra-articularly. Reduced drain output indicates a reduction in hemarthrosis, which could help relieve pressure on the wound and reduce wound problems [9]. Anti-fibrinolytic drugs like tranexamic acid (TXA) help reduce blood loss, postoperative soakage, seroma, and swelling in patients who have both normal and excessive fibrinolytic responses to surgery and trauma, reducing the risk of postoperative or post-injury problems [10,11,12]. Tranexamic acid is a synthetic lysine derivative with a molecular weight of 157 g/mol that uses a reversible interaction with plasminogen and the active protease, plasmin, to exert its anti-fibrinolytic function [13]. Tranexamic acid can be administered through both intravenous or topical /infiltration routes, and most published research revealed both ways to be effective [13]. At the cellular level, fibrin is constantly deposited and eliminated by fibrinolytic mechanisms. Tranexamic acid inhibits proteolytic degradation of fibrin by blocking the attachment of plasminogen and plasmin [14]. Kwaan et al. [15] have previously shown, in skin wounds, that anti-fibrinolytic agents increase collagen synthesis and tensile strength within granulation tissue, presumably by preserving the fibrin matrix. Additionally, Vinckier et al.'s study concluded that TXA could accelerate normal wound healing by stabilizing the fibrin structures within the non-collapsible dental socket [16].

In this case series, we investigate the effect of TXA in ankle arthroplasty, comparing the results of those who received TXA to the group who did not. We report the blood loss and the postoperative wound complications. This case series has been reported in line with the 'participation responsibility openness commitment experimentation sensitivity sense' (PROCESS) criteria [17].

## Materials and methods

This was a retrospective study that did not require Institutional Review Board/ethics committee approval. Data was collected from the operative theatre records, patient notes, outpatient clinic letters, and the Picture Archiving and Communication System (PACS) version 6 (Centricity, GE Healthcare, Chicago, USA) for image evaluation. Patients with incomplete clinical records were excluded from the study. A retrospective analysis was conducted on 69 consecutive patients who underwent TAR surgeries with the Integra® and Cadence® total ankle prosthesis between September 2014 and December 2019. All surgeries were performed at one institution by two, foot and ankle surgeons through the anterior approach. Intraoperatively, TXA was given before tourniquet inflation. The surgeons did not use any form of surgical drains. We recorded patients’ demographics, co-morbidities, type of anaesthesia, dose of TXA, preoperative and postoperative hemoglobin levels, indications for surgery, surgical procedures, wound complications, and postoperative complications.

To assess functional outcomes, the Manchester-Oxford foot questionnaires (MOXFQ) were recorded perioperatively and postoperatively. The MOXFQ patient-reported outcomes (PROs) are a set of 16 self-administered, paper-based assessments [18]. Walking/standing, pain, and social interaction are the three domains measured by the PROs. Patients react on a five-point Likert scale ranging from no limitation to extreme limitation. Each domain’s score is derived by adding the replies to each item within that domain. The raw scores can be translated to a 0 to 00 scale, with 100 being the most significant. All complications were documented. Wound infection, deep infection, aseptic loosening, malalignment, nerve or tendon injury, venous thromboembolism, and periprosthetic fracture are significant problems. Minor complications were noted, including stiffness, loose bodies, and heterotopic ossification. Our definition of revision is “any surgery leading to exchange or removal of any prosthetic component except for the incidental exchange of the polyethylene insert in a movable bearing (three-component) ankle replacement,” as proposed by Henricson et al. [19].

The statistical package for social science (SPSS) version 24.0 (IBM Corp., Armonk, NY, USA) was used to analyze demographic data with descriptive statistics.

## Results

Sixty-nine patients with complete records were included in the study, including 31 females and 38 males. Thirty-three received TXA, while 36 did not. The mean age was 67.2 years (standard deviation (SD)=10.16). Indications of surgery were recorded as failed fusion in five patients, post-traumatic arthritis in 18 patients, and rest as osteoarthritis. Cadence implants were used in 43 patients, and 26 had Integra TAR. The anesthetics varied between general and spinal, and the popliteal block was recorded in only six patients. The dose of TXA varied between 1 gm and 2 gm. The preoperative and postoperative hemoglobin (Hb) and wound complications are reported in Table 1 and Table 2. None of the patients required blood transfusion postoperatively. No cases of deep venous thrombosis or pulmonary embolisms were reported.

**Table 1:** Preoperative and postoperative hemoglobin TXA: Tranexamic acid, SD: Standard deviation

	Preoperative Hb	Postoperative Hb
TXA	138.3 (SD 11.9)	128.1 (SD 9.87)
Non-TXA	138.6 (SD 13.8)	127 (SD 15.5)
P-value	0.9236	0.7289
95% Confidence interval	-6.5186 to 5.9186	-5.2093 to 7.4093

**Table 2:** Patients who developed wound complications OA: Osteoarthritis, GERD: Gastroesophageal reflux disease, GA: General anesthesia, NPWT: Negative pressure wound therapy, HTN: Hypertension, NIDDM: Non-insulin-dependent diabetes mellitus, DM: Diabetes mellitus, COPD: Chronic obstructive pulmonary disease

Age/Sex	PMH	Anesthetics	TXA	Preop Hb	Postop Hb	Indication	Postop complication	Management
63/M	OA	GA	No	140	128	OA	Delayed wound healing	Observed
63/M	Depression	Spinal	No	122	116	Post-traumatic OA	Delayed wound healing	Observed
76/F	HTN, NIDDM, Hypothyroid	Spinal	No	139	124	OA	Deep Infection	Washout/IV antibiotics
52/M	DM	GA	No	174	173	OA, Failed fusion	Superficial infection	Oral antibiotics
70/M	GERD	Spinal	No	149	131	Post-traumatic OA	Delayed wound healing	Observed
62/F	COPD, Smoker	Spinal	No	136	120	Post-traumatic OA	Superficial infection	Oral antibiotics
56/F	Asthma	GA	No	136	118	OA	Wound dehiscence	NPWT
40/F	Fit and well	GA	No	126	120	Post-traumatic OA	Wound dehiscence	Observed
57/F	Learning difficulty	GA	Yes: 1 gm	133	122	OA	Wound dehiscence	Observed
75/M	OA	Spinal	Yes: 2 gm	131	127	OA	Wound dehiscence	NPWT
77/M	Fit and well	Spinal	Yes: 1.5 gm	143	120	OA	Superficial infection	Oral antibiotics

Four cases of wound infection were reported in this series which represents 5.8%. Only one patient (1.4%) required a formal washout in the theatre. Three patients out of 33 TXA patients (9%) developed wound complications. There were two cases of wound dehiscence, one of which required negative pressure wound therapy (NPWT). One (3%) developed superficial wound infection, which responded to oral antibiotics.

**Figure 1:**
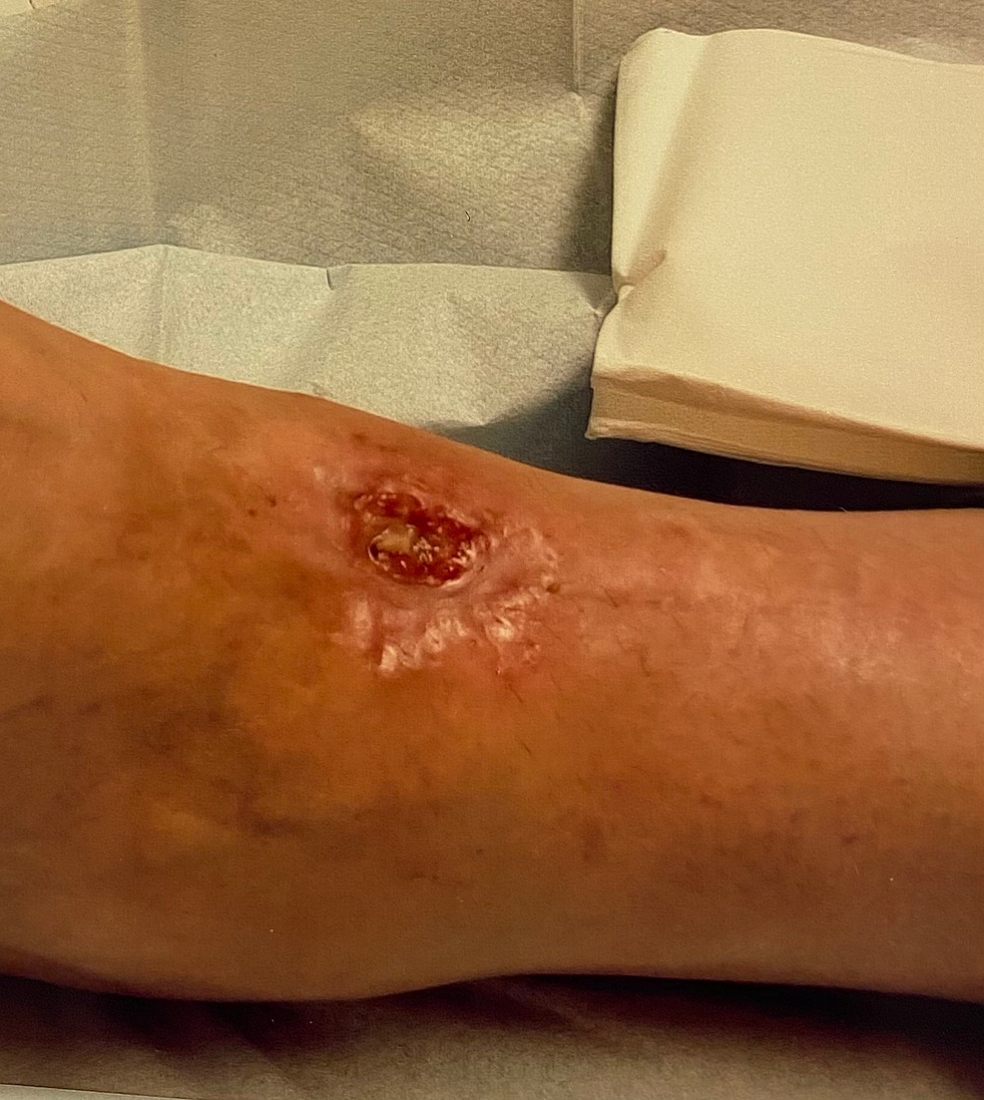
Wound dehiscence requiring negative pressure wound therapy

Eight out of 36 non-TXA patients (22%) developed wound complications. Three (8.3%) had delayed wound healing, one deep infection had formal washout (2.8%), two superficial (5.6%) wound infection cases responded to oral antibiotics, and two cases (5.6%) of wound dehiscence, one of which required NPWT (Figure 1). The Chi-square tests revealed a significant statistical difference between the two groups (p=0.002) when comparing the incidence of wound complications.

The preoperative mean MOXFQ for all patients was 85.35 (SD±6.31) which was significantly improved to 18.15 (SD±13.64). The TXA group achieved better results compared to the non-TXA group (p=0.0130). The results of pre and postoperative MOXFQ for each group are reported in Table 3.

**Table 3:** Results of MOXFQ scores MOXFQ: Manchester-Oxford foot questionnaire, SD: Standard deviation, TXA: Tranexamic acid

	Preoperative MOXFQ (SD)	Postoperative MOXFQ (SD)
TXA	86.31 (SD±5.33)	13.96 (SD±11.17)
Non-TXA	84.46 (SD±6.98)	21.98 (SD±14.55)
P-value	0.2234	0.0130
95% Confidence interval	-4.8548 to 1.1548	1.7447 to 14.2953

## Discussion

Our study assessed the effect of TXA in ankle arthroplasty. There was no staconcerningnificant difference with regard to blood loss (p=0.728). However, there was a significant effect on wound complications (p=0.002). The TXA group reported three patients with wound complications while there were eight patients in the non-TXA group. Furthermore, the TXA group reported significantly better outcome scores following TAR (p=0.013).

Total ankle replacement surgery has become an effective management option with more favorable results than ankle fusion [1]. Furthermore, recent advances in prosthetic designs have significantly improved the survival and short to midterm clinical and radiological outcomes. On the other hand, wound complications and surgical site infections remain major concerns with rates ranging from 0% to 13%, with diabetes, autoimmune diseases, smoking, and coronary and peripheral vascular disease as risk factors [4,5]. The ankle, when compared to hips and knees, has a thin, soft tissue coverage with no elaborate elastic properties [9,20]. Thus, the resultant swelling from intraoperative soft-tissue release and the bleeding from bone and blood vessels are not well accommodated by the small intra-articular volume [20].

Although using a tourniquet in TAR will significantly reduce intraoperative blood loss, the internal bleeding after releasing the tourniquet comes with an increased risk of postoperative swelling, pain, and wound complications. Furthermore, patients with no arterial disease usually have a rich vascular supply surrounding the ankle, and these blood vessels do not tolerate dislocation and subluxation, as in the case of THA or TKA [21]. Hence the shear forces can easily tear the branches of the anterior tibial, causing more bleeding and swelling [22]. Therefore, reducing hemarthrosis within the ankle joint may lead to a decrease in postoperative swelling, decreased pain, and increased range of motion due to the diminished potential for fibrosis [23]. 

Currently, most hip and knee surgeons are routinely using TXA to minimize the amount of intraoperative bleeding. Yuan et al. conducted a study in 2014 to see if TXA could heal the skin barrier using injured skin models and tight intercellular connections. They concluded that TXA might speed skin barrier healing and upregulate occludin induced by physicochemical damage to human skin using bioengineering technologies and immunohistochemical assays [24]. Björlin et al. [25] studied the effect of epsilon-aminocaproic acid and TXA on wound healing in white rats. Wounds injected with TXA had higher tensile strength than those pre-treated with epsilon-aminocaproic acid. Even when compared with the same ionic strength, similar results were obtained. Guerreiro et al. investigated the role of TXA in total knee arthroplasty in a randomized control trial [26]. Before the joint capsule was closed, 22 patients were given topical TXA. In the initial hours after introducing TXA, decreased pain and boosted flexion gain in addition to a reduction in bleeding were noted. Even though particular research revealed that TXA might have adverse effects on tendon repair [27], some authors advocated for TXA's beneficial aspects on wound healing and skin regeneration. Björlin et al. highlighted that TXA has a good impact on the healing process and this effect is not due to the anti-fibrinolytic properties, as both are potent fibrinolytic inhibitors.

In addition, Gupta et al. [28] looked at the role of TXA in reducing wound infection in orthopedic patients after surgery. A total of 120 surgeries were included in the study, including spine, intertrochanteric fractures, hip hemiarthroplasty, and general trauma. Two of the 60 patients on TXA became infected, while four of the 60 patients who were not on TXA became infected. In their series, Mannan et al. [13] concluded that TXA improves hemostasis after deflating the tourniquet, and decreases the swelling and wound complications postoperatively. They also suggested that TXA is effective in reducing wound dehiscence rate, and the need for prolonged antibiotics. As elbows too are similar to ankles as they have thin, soft tissue coverage and small intra-articular volume.

In this study, we observed a significant reduction in postoperative wound complications in patients who received TXA. At the same time, there was no statistically significant difference in blood loss, transfusion rates, or incidence of pulmonary embolisms and deep venous thrombosis. Although the two groups achieved significant improvement following TAR based on the MOXFQ results, the TXA group’s results were superior. The limitations of this study include its retrospective nature and the relatively small sample size. 

## Conclusions

Tranexamic acid is proven to be safe and effective in reducing postoperative bleeding and maintaining hemostasis after deflating tourniquet, thereby reducing swelling and wound complications like breakdown and dehiscence postoperatively. It has a positive impact on post-operative functional outcomes. Future studies are underway with a bigger sample size evaluating the role of TXA in wound healing after total ankle replacement.
